# Biological Screening of Newly Synthesized BIAN *N*-Heterocyclic Gold Carbene Complexes in Zebrafish Embryos

**DOI:** 10.3390/ijms161024718

**Published:** 2015-10-16

**Authors:** Muhammad Farooq, Nael Abu Taha, Rachel R. Butorac, Daniel Anthony Evans, Ahmed A. Elzatahry, Elsayed Ahmed Elsayed, Mohammad A. M. Wadaan, Salem S. Al-Deyab, Alan H. Cowley

**Affiliations:** 1 Bioproducts Research Chair, Department of Zoology, College of Science, King Saud University, Riyadh 11451, Saudi Arabia; E-Mails: nabutaha@ksu.edu.sa (N.A.T.); eaelsayed@ksu.edu.sa (E.A.E.); wadaan@ksu.edu.sa (M.A.M.W.); 2 Department of Chemistry, the University of Texas at Austin, Austin, TX 78712, USA; E-Mails: rrbutorac@utexas.edu (R.R.B.); daevans@utexas.edu (D.A.E.); acowley@cm.utexas.edu (A.H.C.); 3 Materials Science and Technology Program, College of Arts and Sciences, Qatar University, PO Box 2713, Doha, Qatar; E-Mail: aelzatahry@qu.edu.qa; 4 Natural and Microbial Products Department, National Research Centre, Dokki, Cairo 12311, Egypt; 5 Petrochemical Research Chair, Department of Chemistry, King Saud University, Riyadh 11451, Saudi Arabia; E-Mail: ssdeyab@ksu.edu.sa

**Keywords:** BIAN, *N*-heterocyclic carbenes complexes, zebrafish, oxidative stress, neurotoxicity

## Abstract

*N*-Heterocyclic carbene (NHC) metal complexes possess diverse biological activities but have yet to be extensively explored as potential chemotherapeutic agents. We have previously reported the synthesis of a new class of NHC metal complexes *N*-heterocyclic with acetate [IPr(BIAN)AuOAc] and chloride [IPr(BIAN)AuCl] ligands. In the experiments reported herein, the zebrafish embryos were exposed to serial dilutions of each of these complexes for 10–12 h. One hundred percent mortality was observed at concentrations ≥50 µM. At sub-lethal concentrations (10–30 µM), both compounds influenced zebrafish embryonic development. However, quite diverse categories of abnormalities were found in exposed embryos with each compound. Severe brain deformation and notochord degeneration were evident in the case of [IPr(BIAN)AuOAc]. The zebrafish embryos treated with [IPr(BIAN)AuCl] exhibited stunted growth and consequently had smaller body sizes. A depletion of 30%–40% glutathione was detected in the treated embryos, which could account for one of the possible mechanism of neurotoxicity. The fact that these compounds are capable of both affecting the growth and also compromising antioxidant systems by elevating intracellular ROS production implies that they could play an important role as a new breed of therapeutic molecules.

## 1. Introduction

*N*-Heterocyclic carbene (NHC) metal complexes are easy to synthesize and functionalize since they form stronger bonds to metals and therefore form more stable metal complexes [1,2]. The *N*-heterocyclic carbene (NHC) metal complexes also possess diverse biological activities and a few of them have been reported as potential chemotherapeutic agents [3,4,5,6,7,8,9]. The cationic Au(I) complexes of *N*-heterocyclic carbenes (NHCs) have been reported to have dual properties in a single molecule by targeting the mitochondria of cancer cells and selective inhibition of thioredoxin reductase [10,11,12,13]. The anti-mitochondrial properties of cationic, linear Au(I) *N*-heterocyclic carbene complexes have also been reported in a different study [14]. Even though the gold *N*-heterocyclic carbene complexes have been shown to have great commercial value for medical applications, there is often a lack of relevant safety and toxicology data. Most of the toxicological studies have been focused on *in vitro* models, which cannot represent the safety assessment in an entire organism. Toxicological studies in *in vivo* systems have greater significance over *in vitro* systems related to diversity in physiology and anatomy.

Zebrafish represent an excellent *in vivo* model that is routinely used to test the *in vivo* toxicity of small compounds [15,16,17]. We have previously reported the synthesis of bis(imino)acenaphthene (BIAN)-supported *N*-heterocyclic carbene (BIAN N-NHC) complexes of gold *N*-heterocyclic and their antimicrobial and antifungal activities [18,19]. The objective of this study was to test the bioactivity and *in vivo* toxicity of these *N*-heterocyclic complexes in zebrafish embryos.

## 2. Results

### 2.1. BIAN N-Heterocyclic Carbene Gold Complexes Induce Lethality and Malformations in Zebrafish Embryos

**Figure 1:**
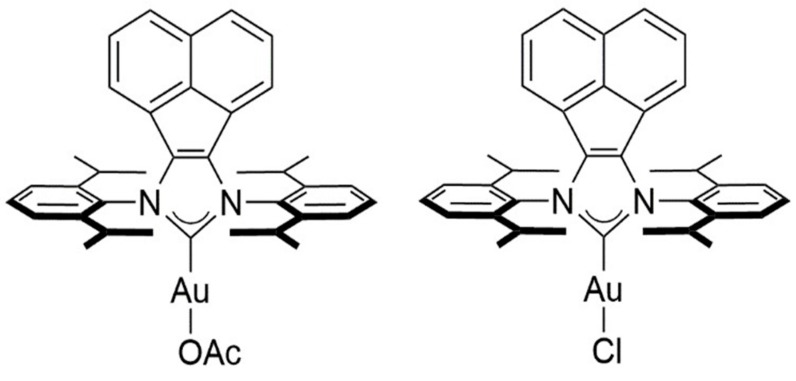
Structures of the BIAN N-NHC gold complexes.

The structures of the newly synthesized BIAN *N*-heterocyclic carbene (NHC) complexes are given in Figure 1. The detailed synthesis procedure has been reported earlier as described in the experimental section.

Wild-type zebrafish embryos were exposed to gold acetate [IPr(BIAN)AuOAc] and gold chloride [IPr(BIAN)AuCl] complexes within the concentration range of 1–100 µM. Both compounds were safe in zebrafish embryos up to a concentration of 10 µM (Table 1) and no obvious abnormality was noticed in exposed embryos by either compound. A range of mortality (10%–60%) was observed when the embryos were exposed to concentrations between 30–50 µM. Concentrations higher than 50 µM were lethal and 100% mortality was observed with both compounds. Generally, the gold acetate complex [IPr(BIAN)AuOAc] turned out to be more toxic than the corresponding gold chloride [IPr(BIAN)AuCl] complex at the same molar concentration (Table 1).

**Table 1:** Mortality of zebrafish embryos upon exposure to a range of concentrations of the BIAN-NHC gold carbene complexes.

Concentration (µM)	[IPr(BIAN)AuCl]		[IPr(BIAN)AuOAc]	
	Number of Embryos Used *	% Mortality	Number of Embryos Used *	% Mortality
Control (1% DMSO *v*/*v* treated embryos)	54.00 ± 0.57	0.0 ± 0.00	50.00 ± 0.57	0.0 ± 0.00
1	51.00 ± 0.59	0.0 ± 0.00	51.00 ± 0.57	0.0 ± 0.00
10	50.00 ± 1.15	1.0 ± 1.00	50.00 ± 1.00	3.0 ± 0.5
15	50.00 ± 1.15	3.0 ± 1.00	50.00 ± 1.52	5.0 ± 0.50
30	51.00 ± 1.15	5.0 ± 0.57	51.00 ± 1.52	10.0 ± 1.14
50	52.00 ± 1.15	25.0 ± 1.52	52.00 ± 1.15	60.0 ± 2.00
100	50.00 ± 1.00	80.0 ± 0.57	50.00 ± 1.16	100 ± 0.00

* Mean value from three independent experiments ± SD.

### 2.2. The Gold Acetate Complex [IPr(BIAN)AuOAc] Induces Neurotoxicity by Hindering Brain Formation and Degeneration of the Notochord in Exposed Embryos

**Figure 2:**
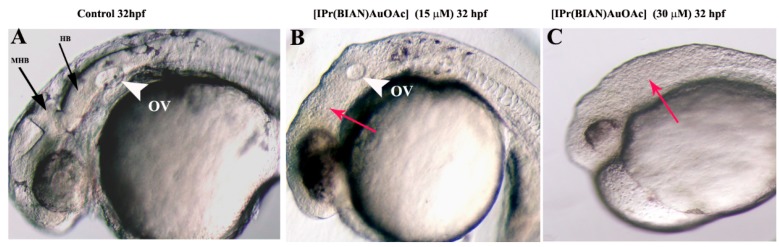
BIAN NHC gold complexes affected the brain development in treated zebrafish embryos. Representative images of zebrafish embryos exposed to 50 µM of gold acetate, gold chloride and mock treated (1% DMSO *v*/*v*) embryos at 32 h post fertilization (hpf). The black arrows indicate the prominent mid–hindbrain boundaries between the posterior midbrain and the anterior hindbrain in the control embryos (**A**); the red arrow signifies the absence of clear mid-hindbrain boundaries between the posterior midbrain and the anterior hindbrain after exposure to the gold acetate compound (**B**) and the gold chloride compound (**C**). The otic vesicle (OV) was not developed in the gold acetate treated embryos. Abbreviations used: MHB = midbrain hindbrain boundary, HB = hindbrain, and OV = otic vesicle.

Head hypoplasia (small head) was found to be the most prominent phenotype for zebrafish embryos that were exposed to [IPr(BIAN)AuOAc]. Normal brains with distinct morphological boundaries are clearly evident in the control embryos at 32 h post fertilization (hpf) (Figure 2A). The treated embryos, on the other hand, featured smaller heads with unclear boundaries between the brain subdivisions. These effects were particularly obvious in the mid–hindbrain region (Figure 2B,C). Moreover, the mid-hindbrain boundary, which was prominent in the case of the control embryos (Figure 2A), was not obvious in the treated embryos (Figure 2B,C). A dose dependent severity of head hypoplasia was noticed in the exposed embryos. The incidence of head hypoplasia was 100% (*n* = 200 ± 28.86) at 30 µM concentration, while only 60% of the embryos (*n* = 210 ± 15.27) featured smaller heads after treatment with a 15 µM concentration of [IPr(BIAN)AuOAc] (Figure 2B,C). Furthermore, the otic vesicle (equivalent to the human ear) was significantly smaller in comparison with control at 15 µM exposure, while the otic vesicle was not evident in the case of the 30 µM treated embryos (white arrowhead in Figure 2A,B).

**Figure 3:**
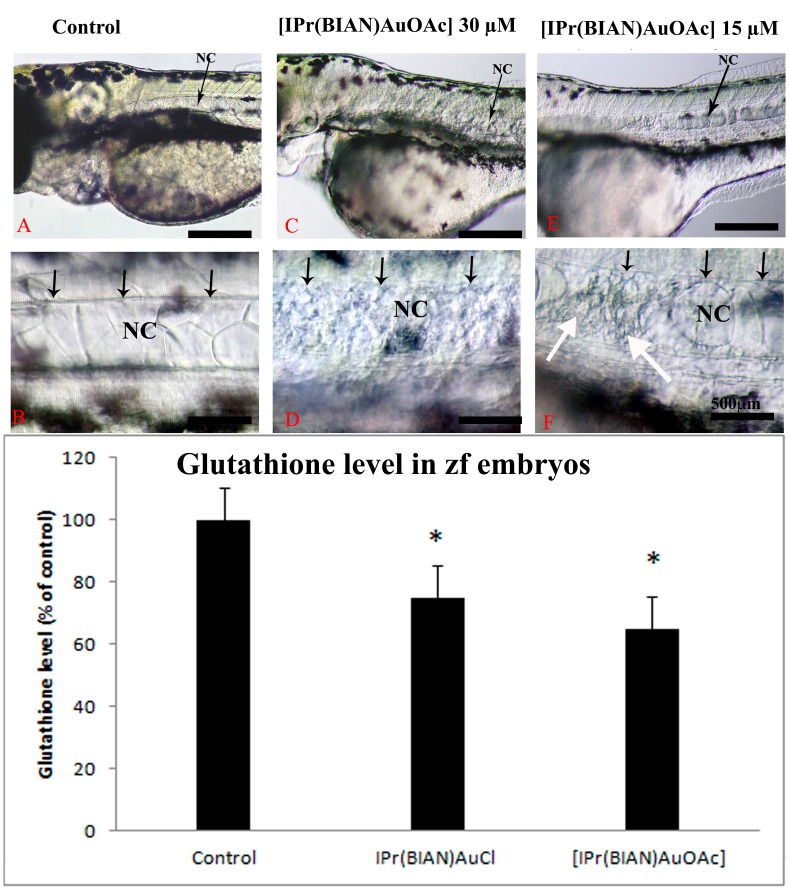
BIAN NHC Gold Complexes induced degeneration of the notochord in treated zebrafish embryos by inducing oxidative stress. **Upper panel**: Live photomicrographs of representative zebrafish embryos that had been treated with mock (1% DMSO *v*/*v*) (**A**,**B**); 30 µM of [IPr(BIAN)AuOAc] (**C**,**D**) and IPr(BIAN)AuCl 50 µM (**D**,**F**) at three dpf. The notochord (represented as NC in the images) in the mock treated embryos lies flat and is located between the organized layers of the sheath cells (indicated by black arrows). In contrast, the embryos that were treated with [IPr(BIAN)AuOAc] exhibited complete degeneration of the entire notochord (see black arrows in **D**); It is also noteworthy that the distinct boundaries between the sheath layers are absent in the case of the treated embryos (Compare black arrows in **B**,**D**); The [IPr(BIAN)AuCl] treated embryos also exhibited degeneration of the notochord, but in a somewhat localized manner, mostly at the anterior trunk (white arrows in **F**); The sheath cells were also normal in the case of the [IPr(BIAN)AuCl] treated embryos (black arrows **F**); **Lower panel**: glutathione (GSH) levels in zebrafish embryos exposed for 24 h. Each histogram represents the mean ± SD of three independent experiments. * *p* < 0.05 *vs.* control.

The zebrafish embryos that had been exposed to 30 µM of [IPr(BIAN)AuOAc] exhibited degeneration of the notochord. It was noticed that the formation of the notochord was not interrupted in the [IPr(BIAN)AuOAc] exposed embryos. However, the notochord degenerated after it was formed. The deterioration of the notochord was more obvious at 72 hpf (Figure 3). The notochord cells lie completely within the sheath cells in the control embryos (Figure 3A; notochord represented by NC), whereas the notochord cells of the embryos that were treated with [IPr(BIAN)AuOAc] exhibited fragmented cells (Figure 3C,D, white arrows) throughout the notochord along with disorganized sheath cells (Figure 3C,D, black arrows). The severity of degeneration in the notochord was also dose dependent. At 15µM concentration the degeneration occurred in a somewhat localized manner, mostly at the anterior trunk (Figure 3E,F, white arrows) and the sheath cells were also normal and straight (Figure 3E,F, black arrows). The degeneration of the notochord became severe in the 30 µM treated embryos and deteriorated notochord cells were prominent throughout the entire trunk along with disorganized sheath cells (Figure 3C,D, white arrows).

The presence of oxidative stress could lead to neurodegeneration. In order to check whether these compounds induced any oxidative stress in exposed zebrafish embryos, the level of total glutathione (GSSG + GSH) in the treated embryos was measured. A 40% reduction in the total glutathione was detected in the zebrafish embryos that had been treated with 30 µM of the gold acetate complex [IPr(BIAN)AuOAc] while the gold chloride complex [IPr(BIAN)AuCl] reduced the glutathione by 25% at 30 µM (Figure 3) lower panel.

### 2.3. The BIAN NHC Gold Chloride Complex [IPr(BIAN)AuCl] Exposure Induced Delay in Development, Stunted Growth and Organ Malformation in Zebrafish Embryos

**Figure 4:**
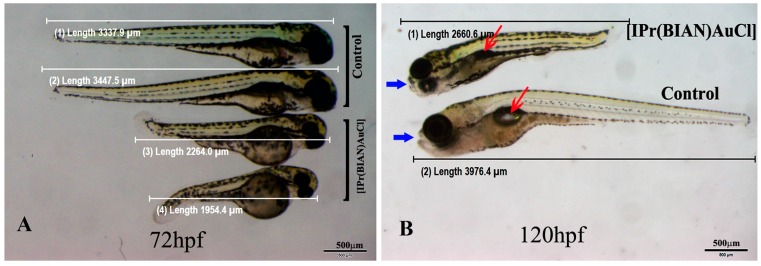
The [IPr(BIAN)AuCl] exposure induced delay in development decrease growth and organ malformation in zebrafish embryos. Representative live images of the control and treated zebrafish embryos at three dpf (**A**); and five dpf (**B**). The body size comparison of the mock treated embryos (1% DMSO *v*/*v*) and the 30 µM [IPr(BIAN)AuCl] treated embryos at three dpf revealed that the latter treated embryos were significantly smaller in size. The swim bladder (red arrow **B**) is evident in the control embryo but fails to form in the case of the treated embryos (red arrow). The mouth structure snout (blue arrow head) also did not form in treated embryos. The treated embryos failed to grow to normal size and remained small when observed at five dpf. In order to avoid bias, the images were captured by placing treated and untreated embryos side by side at the same magnification).

The zebrafish embryos exposed to 30 µM concentrations of [IPr(BIAN)AuCl] failed to grow and remained smaller as compared with the untreated control embryos (Figure 4A). Careful body measurements revealed that the mean body lengths from the head to the tip of tail of the untreated embryos (mean number of embryos 30 ± 1.6) were 0.3939 ± 0.002 cm, while the mean body lengths of the treated embryos (mean number of embryos 30 ± 1.7) were 0.2168 ± 0.0049 (*p* values 2.72625 × 10^‒53^) which is almost 62% smaller than that of the untreated or mock (1% DMSO *v*/*v*) treated embryos. In order to check, whether these short bodied embryos could regain the normal size if they were raised without compound after a short exposure. The embryos were exposed to [IPr(BIAN)AuCl] for 12 h and subsequently raised in a compound free medium for another 96 h. Interestingly, these short bodied embryos failed to reach to normal size (Figure 4B). The exposed embryos also had malformed organs. For example the control embryos had normal swim bladders (shown by red arrows in Figure 4B), whereas the exposed embryos did not develop swim bladder. The exposed embryos also had malformed snouts (blue arrow head in Figure 4B).

**Figure 5:**
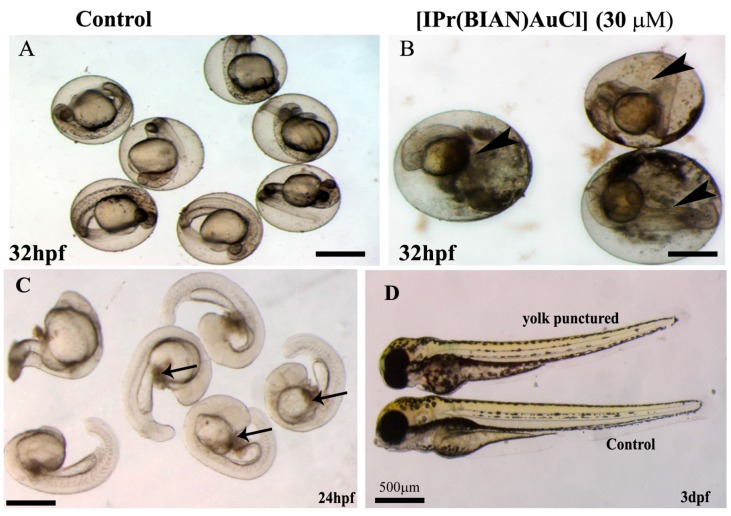
The shortened bodies that were observed by treatment with the BIAN NHC gold chloride complex [IPr(BIAN)AuCl] were not due to the leakage of yolk contents. The control embryos at 32 hpf had intact yolks and there was no leakage of the yolk content (**A**); However, leaky yolk was evident in the zebrafish embryos that had been treated with the BIAN NHC gold chloride complex [IPr(BIAN)AuCl] 30 µM (black arrow head in **B**) and this could be the reason for the shortened bodies; In order to validate this hypothesis, the yolks of the untreated embryos were punctured at 24 hpf (**C**, black arrows); and these embryos were allowed to grow until three dpf. Even though there was some developmental delay for the embryos that had been punctured, they grew to the same size as the control (compare the top embryos to **D**). The images shown in (**D**) were taken using the same magnification by placing the embryos side by side.

The mechanism by which the [IPr(BIAN)AuCl] affected the growth of zebrafish embryos is unknown. A smaller yolk and leakage of yolk contents was observed in the case of the exposed embryos (Figure 5B). It was suspected that un-availability of nutrients due to the leakage of yolk might be the reason for the stunted growth. The yolk is rich in nutrients and considered to be the primary source of nutrition for zebrafish embryos for four to five days. In order to test this hypothesis, the untreated zebrafish embryos were raised up to 24 hpf, following which the yolk was punctured using sterile 1cc syringe needles (Figure 5C) and allowed to grow up to 72 hpf. Most (12/15) of the embryos died when the yolk was punctured. However, the embryos that survived had no significant difference in body lengths as compared with that of the control (Figure 5D). This implies that malnutrition was not the exclusive reason for the stunted embryonic growth and that [IPr(BIAN)AuCl] could have influenced the embryonic growth by interfering with the overall cell proliferation and growth.

## 3. Discussion

Anticancer drugs that are widely used have significant side effects such as neurotoxicity, nephrotoxicity, and the development of resistance in some cancer cells [20]. In order to overcome these limitations, a variety of different metal-containing complexes are currently being studied as potential anticancer agents [21]. Among such compounds, the metal *N*-heterocyclic carbene (NHC) complexes are highly stable and easy derivatization makes them suitable candidates for drug development [22,23]. Of the various metal NHC complexes that have been prepared thus far, the silver and gold NHC complexes show considerable promise as anti-microbial and anticancer agents [24]. At the present time, the biomedical applications of gold *N*-heterocyclic carbene are in their infancy, and the available literature is limited to only a few reports [25,26,27,28,29]. There is very little data on the effects of the gold *N*-heterocyclic carbene complexes (and other metallic heterocyclic carbene complexes) on zebrafish embryonic development, with only two studies having addressed the mortality of zebrafish embryos with respect to ruthenium based *N*-heterocyclic carbene complexes. The results of these studies are also contradictory: in one study the ruthenium-*N*-heterocyclic carbene complexes were reported to be harmless to zebrafish embryos under all tested conditions, [28] while, in the other, greater than 50% mortality was reported following exposure of the embryos to ruthenium(II) *N*-heterocyclic carbene complexes for 48 h [29]. This striking disparity suggests that the lethality or toxicity of a tested complex depends primarily on the nature of the transition metal that is incorporated in the *N*-heterocyclic carbene complex. For example, in the studies described above, the ruthenium(II) benzimidazolylidene carbene complexes were found to be more toxic than the benzimidazolylidene carbene free ruthenium complexes. Bearing this, and the above discussion in mind, the bioactivity of the BIAN *N*-heterocyclic gold carbene complexes in zebrafish embryos could be due to structural modification. In particular, the addition of an “acetate” group could have contributed to the overall toxic character. In fact, the presence of various combinations of acetate groups have been shown to induce mortality in exposed zebrafish embryos [30,31]. The high mortality in response to the gold acetate complex [IPr(BIAN)AuOAc] that was encountered in the present study is clearly in accord with results reported for other studies [30]. Moreover, ruthenium and gold *N*-heterocyclic carbene complexes have been reported to be toxic to other animal models as well [26,27].

In the present study the gold acetate complex [IPr(BIAN)AuOAc] targeted the neurogenesis in zebrafish embryos either by impeding brain formation or causing the degeneration of notochord. The precise mechanisms by which this compound impaired the neurogenesis in zebrafish embryos are not known. However, one candidate mechanism is oxidative stress, since this is usually regarded as the major contributor to neurodegeneration and CNS injury [32,33,34,35]. Oxidative stress can also induce neuronal cell death and is considered a common pathway in various forms of neuronal cell death in neurological diseases [36,37].

In an oxidative stress state the intracellular glutathione decreases with an elevated level of oxygen-derived free radicals [38,39,40]. Glutathione is a critical component of antioxidant defense and has been linked directly to oxidative stress. Glutathione plays an important role in protecting neurons against oxidative damage and metabolic insults by detoxifying 4-hydroxynonenal (HNE) [41]. A depletion of glutathione caused nerve cell death in immature cortical neurons and a neuronal cell line [42]. The neurotoxicity and notochord neurodegeneration was noticed in zebrafish embryos that had been exposed to [IPr(BIAN)AuOAc], and consequently 40% reduction in the total glutathione level was found in treated embryos. This reduction of glutathione might have elevated the oxidative stress in the exposed embryos. Hence the oxidative stress could be one of the mechanisms for possible neurotoxicity associated with [IPr(BIAN)AuOAc] in zebrafish embryos. The generation of ROS in cultured tumour cells by two species of gold(I) NHC complexes has been reported [43,44], but there is no report so far, about the status of oxidative stress in live animals by exposure to any of the gold NHC complexes.

The literature about the growth inhibition and reduced body size that has been observed in zebrafish embryos exposed to [IPr(BIAN)AuCl] is very limited. Only one report described a somewhat similar phenotype. Titanium dioxide nanoparticles (TiO_2_NPs) produced similar kind of stunted growth, delayed metamorphosis, malformations, organ pathology, and DNA damage in exposed zebrafish larvae. The induction of reactive oxygen species (ROS) was believed to be the mechanism that caused toxicities in zebrafish embryos by titanium dioxide nanoparticles (TiO2NPs) [45]. The [IPr(BIAN)AuCl] exposure also induced a 30% reduction in the total glutathione level which in turn could have compromised the oxidative status in exposed zebrafish embryos. In agreement with the above mentioned study, the oxidative stress could possibly be one of the mechanisms for growth inhibition in [IPr(BIAN)AuCl] embryos.

The development of organs shares many similarities with cancer development in terms of biological behaviour and molecular basis [46,47]. This means that if a certain type of molecule(s) interrupt the formation of an organ during embryonic development by suppressing the expression of crucial transcription factors which are needed for cell proliferation, it is quite likely that the same set of compounds would target those transcription factors in pathological states as well. Moreover, very recently, conserved gene expression was identified by comparing oncogenesis in the human cerebellar tumour, MB, and the developing wild-type mouse cerebellum during postnatal days 1–60 [48]. In our experiment, the BIAN *N*-heterocyclic gold carbene complexes specifically interrupted brain formation so it is very important to find out the molecular target(s) affected after the exposure of these compounds in zebrafish embryos in order to predict the therapeutic application of these molecules in respect to human brain tumours. Our unpublished results with cancer cells [49] have revealed that both of these compounds have shown cytotoxicity towards two types of human cancer cells line namely liver carcinoma (HepG2) and breast carcinoma (MCF7). Both compounds affected the viability of cancer cells with IC_50_ values less than micro molar range. However, these compounds did not induce any kind of abnormalities in zebrafish embryos up to a 10 µM range. This means that at low concentrations (1–10 µM) they might be cytotoxic to cancer cells but safe to be used in animals (at least in the case of zebrafish embryos) which in turn suggests the potential use of these compounds as promising therapeutics drugs.

## 4. Experimental Section

### 4.1. Biological Screening

#### 4.1.1. Animals

Wild type (AB/Tubingen tab-14) zebrafish were obtained from the Zebrafish International Resource Center (ZIRC University of Oregon, Eugene, OR, USA). Wild type zebrafish were kept under the standard laboratory conditions of 28 °C on a 14 h light/10 h dark photoperiod in fish water, consisting of reverse osmosis water supplemented with a commercially available salt (0.6% Instant Ocean salt). All the embryos used in these experiments were less than 120 h post fertilization and hence do not need the approval of Animal Care and Use Committee as stated by Strahle [49]. However, all the experiments were carried out in accordance with the International Animal Use Guidelines.

#### 4.1.2. Embryo Treatment

Embryos were obtained by natural pairwise mating and were raised up to the shield stage (6 h post fertilization). The gold complexes were re-suspended in cell culture grade dimethyl sulfoxide (DMSO) (D8418 Sigma LLC. St. Louis, MO, USA) at a stock concentration of 10 mM. The synchronized staged embryos (*n* = 30–35) with chorion were exposed to serial dilutions of the gold complexes in the embryonic media (5.0 mM NaCl, 0.17 mM KCl, 0.33 mM CaCl_2_, 0.33 mM MgSO_4_). The mock controls were 1% DMSO (*v*/*v*) treated embryos. The embryos remained exposed for the indicated times of treatment in an air incubator at 28 °C. This treatment was repeated at least three times using different batches of embryos each time. The cumulative mortalities and morphological defects were assessed at 24, 48, 72, 96 and 144 h post fertilization. The types of malformations that were considered were pericardial oedema (PE), yolk sac oedema (YE), yolk sac malformation (YM), bent spine (BS), brain deformation (BrD), notochord deformation (NC), tail malformation (TM), stunted or delayed growth (SG) and shortened body axes (SB).

#### 4.1.3. Microscopy and Photography

All images were recorded using a Nikon Eclipse E600 Binocular Microscope equipped with a Nikon Digital Camera (Nikon Corporation; Konan, Minato-ku, Tokyo, 108-6290 Japan), model DXM1200F.

#### 4.1.4. Measurement of Glutathione (GSH) Level in Zebrafish Embryos

The changes in the GSH levels in the exposed embryos were determined using a commercially available kit (Glutathione Assay Kit, Catalogue Number CS0260; Sigma- Aldrich 3050 Spruce Street, St Louis, MO, USA). In order to check the total glutathione level in the zebrafish, the embryos were exposed to the gold carbene NHC complexes for 12 h. Following exposure, embryos (both treated and control) were rinsed three times in PBS. The embryos were then homogenized in a cold assay buffer (100 mM potassium phosphate buffer, pH 7.0, with 1 mM EDTA). The resulting homogenate was centrifuged at 10,000 rpm for 15 min at 4 °C, and the supernatant was collected and deproteinated by treatment with 5% 5-sulphosalicylic acid. The total glutathione level was then measured by reading the samples in a plate reader. The amount of glutathione in mock treated embryos was normalized as 100%. The level of glutathione in exposed embryos was calculated as follow:

Total glutathione level = (Mean absorbance in exposed embryos)/(Mean absorbance in control embryos) × 100.

### 4.2. Chemistry

#### Syntheses of BIAN NHC Gold Complexes

The BIAN NHC gold complexes [IPr(BIAN)AuCl] and [IPr(BIAN)AuOAc] were synthesized as reported previously [50].

### 4.3. Statistical Analysis

The data in Table 1 is expressed as mean ± S.D., using the values obtained from three independent experiments. Statistical analysis was performed by one-way analysis of variance (ANOVA) using Origin v6.1052 (Origin Lab Corporation, Northampton, MA, USA). Unless stated otherwise, the chosen level of statistical significance was *p* < 0.05.

## 5. Conclusions

We have previously reported the antimicrobial and antifungal properties of *N*-heterocyclic carbene complexes [18,19]. This study has shown the bioactivity and *in vivo* cytotoxicity of these compounds in an entire organism *i.e.*, zebrafish embryos. All together the antimicrobial, antifungal, anticancer (unpublished data) [48] and elevation of oxidative stress in zebrafish embryos as well as in cancer cells implies that these compounds have the potential to be developed as new class of therapeutics.
